# Stochastic polarity formation in molecular crystals, composite materials and natural tissues

**DOI:** 10.1107/S205225251700700X

**Published:** 2017-05-24

**Authors:** Jürg Hulliger, Matthias Burgener, Rolf Hesterberg, Martin Sommer, Khadidja Brahimi, Hanane Aboulfadl

**Affiliations:** a Department of Chemistry and Biochemistry, University of Bern, Freiestrasse 3, Bern CH-3012, Switzerland

**Keywords:** stochastic polarity formation, molecular crystals, biomimetic materials, natural tissues, Markov chain processes

## Abstract

This topical review summarizes the theoretical and experimental findings obtained over the last 20 years on the subject of growth-induced polarity formation driven by a Markov chain process. When entering the growing surface of a molecular crystal, an inorganic–organic composite or a natural tissue, the building blocks may undergo 180° orientational disorder. Driven by configurational entropy, faulted orientations can promote the conversion of a growing non-polar seed into an object showing polar domains. Similarly, orientational disorder at the interface may change a polar seed into a two-domain state. Analytical theory and Monte Carlo simulations were used to model polarity formation. Scanning pyroelectric, piezoresponse force and phase-sensitive second-harmonic microscopies are methods for investigating the spatial distribution of polarity. Summarizing results from different types of materials, a general principle is provided for obtaining growth-induced polar domains: a non-zero difference in the probabilities for 180° orientational misalignments of building blocks, together with uni-directional growth, along with Markov chain theory, can produce objects showing polar domains.

## Abstract 2

Stochastic polarity formation applies to molecular-based solid matter, in cases where the building blocks undergo 180° orientational disorder at the growing surfaces. The paper reviews progress on molecular crystals, inorganic–organic composites and natural tissues.

## 1. Introduction   

An asymmetric charge distribution (polarity), chirality and the van der Waals shape are the main properties of building blocks that allow nature to build up complex structures and functions. Concerning the solid-state properties of crystals, including less-ordered materials, electric polarity is at the origin of a number of technologically or biologically relevant functions (pyro­electricity, piezoelectricity, optical nonlinearities). For most solids made of neutral organic molecules, these effects originate from molecular properties, modified by the surrounding crystal field of the order of 10^9^ V m^−1^.

The purpose of this topical review is to explain how polarity can build up through processes driving the growth of mol­ecular crystals, biomimetic composite materials and biological tissues. By addressing polarity we focus here only on the directionality, *i.e.* the vectorial alignment of the building blocks, because our physical consideration is based on geometric polarity.

It is found that stochastic processes (Gardiner, 1997 ▸) in general play an essential role in cell biology (Bressloff, 2014 ▸) and that Markov chain theory (Hulliger, 2002 ▸) provides a key to understanding polarity formation during growth and molecular recognition at interfaces. By ‘stochastic’ we understand a system which evolves probabilistically. The Markov concept is expressed in terms of conditional probabilities, being determined by knowledge of the most recent conditions of a system [von Hilgers & Velminski (2007 ▸), a book reviewing Markov’s work and its early development]. In the cases we discuss here, this means that the most recent growth steps on a surface determine how the building blocks (Markov approach for crystal growth; see Gates, 1997 ▸) will contribute to further polar alignment.

In the field of computation, Markov chain Monte Carlo methods became a leading tool in the 1990s, which changed our approach to that of solving complex problems by simulation instead of searching for exact solutions (Robert & Casella, 2011 ▸).

During the last 20 years we have developed a stochastic theory and experimental techniques to elaborate how polarity in three types of material, (i) molecular crystals, (ii) bio­mimetic composite materials and (iii) biological tissues, can evolve during growth (Hulliger, 2002 ▸). For ease of reading it will be helpful to recognize right at the beginning that uni-directional growth combined with orientational selectivity of building blocks can produce small or large polar domains for all the materials we are going to address here.

To study the effects of growth-induced polarity on the scale of millimetres, micrometres and nanometres, new physical techniques are required. Recently, scanning pyroelectric microscopy (SPEM, micrometres to millimetres), piezoresponse force microscopy (PFM, down to a range of 20–50 nm) (for a review of these techniques, see Batagiannis *et al.*, 2010 ▸) and phase-sensitive second-harmonic microscopy (PS-SHM, micrometres to millimetres; Aboulfadl *et al.*, 2013 ▸) have revealed essential features of the polar state of material types (i)–(iii).

In SPEM, a modulated and focused laser diode heats spot-wise a material placed in a capacitor. The displacement current is measured by a lock-in technique. Knowing the current direction allows us to derive the sign of the induced surface charges (+, −). In cases where we know either the absolute structure of a material or the sign of its pyroelectric coefficient, we can derive the direction of the dipolar alignment. By scanning a sample in two dimensions a polarization map is obtained.

In PFM, the tip of an atomic force microscope locally applies an alternative potential very close to the surface of a sample. Due to the converse piezoelectric effect, a surface deformation is induced. In turn, the AFM tip is deflected, thus mapping the local polar properties of the surface.

In PS-SHM we set up an interference experiment. A fundamental laser beam ω_o_ passes through a reference (*R*) nonlinear optical crystal generating 2ω_o_(*R*) light. This wave 2ω_o_(*R*) and the fundamental ω_o_ pass through the sample (*S*). In the sample a second 2ω_o_(*S*) wave is generated according to the spatial distribution of nonlinearity. Using a phase shifter we bring 2ω_o_(*R*) and 2ω_o_(*S*) to constructive interference. In cases where the sample represents a 180° two-domain state, shifting the phase will create second-harmonic light in only one domain, while for the other part the conditions of destructive interference apply (because of *m* or *i* symmetry relating the domains). This allows us to visualize antiparallel domains in two dimensions.

### 1.1. Material type (i): molecular crystals   

The existence of polar structures for molecular crystals is well documented by the Cambridge Structural Database (CSD; Groom & Allen, 2014 ▸). By ‘polar’ we understand here the expression of a point symmetry belonging to one of the ten pyroelectric groups (Nye, 1985 ▸). Only a few crystals made of neutral organic molecules also show ferroelectricity (Choudhury & Chitra, 2006 ▸), *i.e.* a structural phase transition introducing a spontaneous polarization *P*_s_ undergoing inversion by an applied electric field (Blinc, 2011 ▸).

For all these crystals we find a polar axis and (*hkl*), (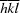) face pairs which permanently carry either a positive or a negative surface charge. Grown under ambient conditions, these faces undergo charge compensation by charge carriers attracted from the environment. To date, no details of the mechanisms of charge compensation have been investigated experimentally for molecular crystals.

**Figure 1:**
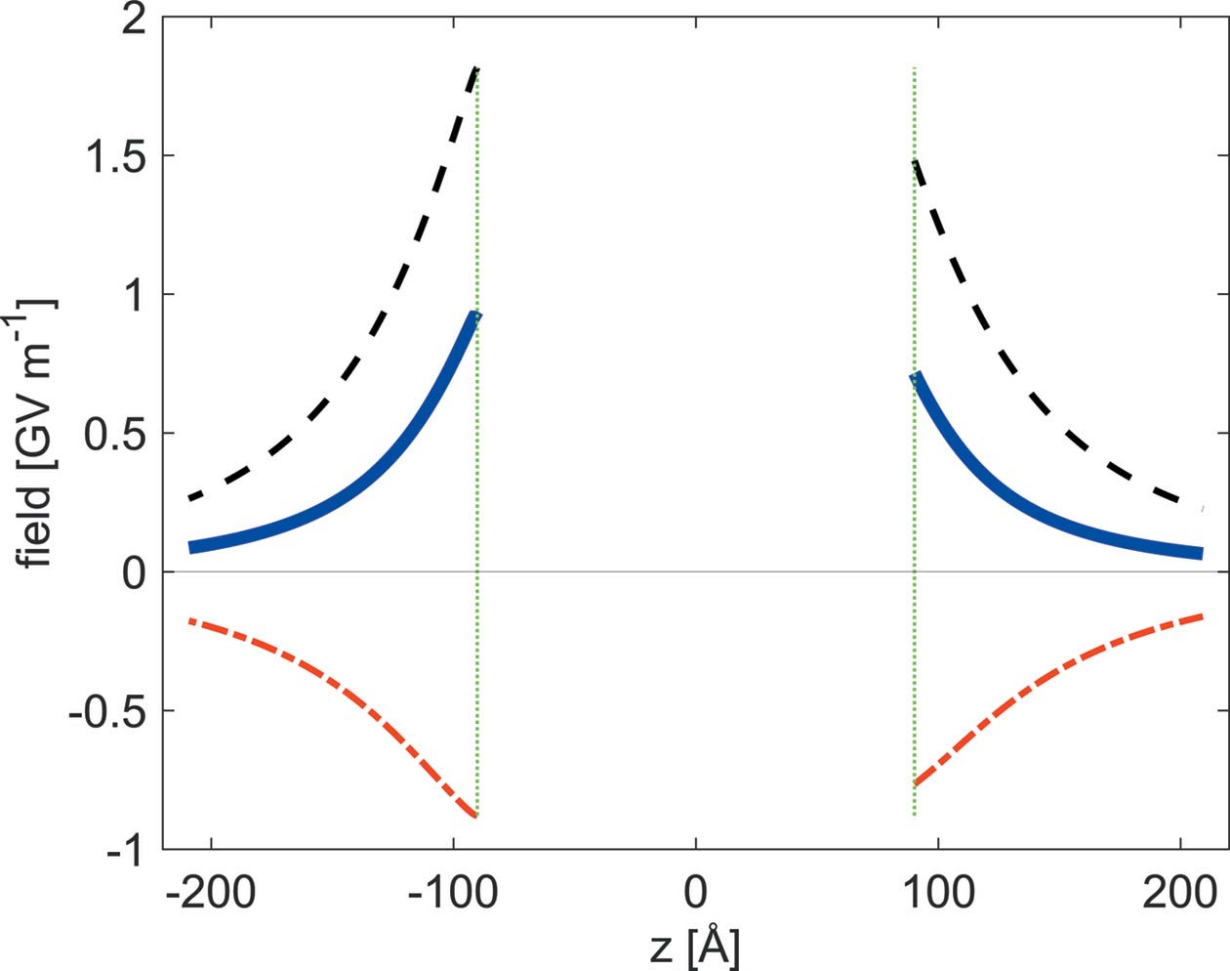
Calculated outer electric field along polar axis 2 of a 4-iodo-4′-nitro-biphenyl (INBP, *mm*2) nano-sized crystal [11 × 11 × 11 unit cells; vertical dotted green lines mark the crystal surfaces (001), (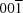)]. Dashed black line: crystal without surface compensation. Dashed red line: compensation field due to a model for added external charges. Blue line: sum of both contributions (Hesterberg *et al.*, 2016 ▸; extended paper to be published).

Recent calculations of the inner and outer electric field for a polar structure [4-iodo-4′-nitro-biphenyl, *Fdd*2 (*mm*2); here, all dipoles are parallel (Sarma *et al.*, 1997 ▸)], including a model for the compensation of surface charge, show (Fig. 1 ▸) that the addition of external charge can reduce the outer field to about 50% compared with the situation featuring charged (001), (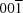) faces. This means that a crystal may preserve a certain macroscopic dipole moment, although its surface charge density is screened to zero (Hesterberg *et al.*, 2016 ▸). Furthermore, these new results demonstrate a surprising shape effect: depending on the relative size (*e.g.* needle *versus* plate), the inner electric field can change its direction. Recently, dipolar enhancement due to the crystal field has been a topic discussed by Spackman *et al.* (2007 ▸). However, present calculations have revealed that one and the same molecule within its lattice can exhibit either an enhancement or a reduction of its dipole moment, depending only on the relative dimensions of the crystal used to measure or calculate the effect. There is no unique answer to this issue.

Let us address a further interesting phenomenon typical of polar crystals where we can recognize a lack of general understanding. For a number of molecular crystals the growth speed along one direction of the polar axis is very slow or almost zero compared with the other direction. Here, experimental and theoretical investigations of the anisotropic growth of α-resorcinol (*mm*2) along the polar axis 2 have so far led to the most advanced view, as follows.

*In situ* kinetic vapor phase measurements demonstrate active growth for the negative side of the axis (where hydroxyl groups are present), whereas growth in the positive direction (H atoms of the benzene ring on the surface) was dependent on the perfection of the surface, although mostly no growth was observed. At elevated supersaturation, a macroscopic roughening took place tending to initiate 180° twinning, ‘… characteristic of the growth of these materials …’ (Srinivasan & Sherwood, 2011 ▸). Similar observations for solution growth have led to the conclusion that ‘… there is sufficient evidence to suggest that the anomalous growth of polar materials is not a consequence of solvent inhibition …’ (Srinivasan & Sherwood, 2005 ▸) and ‘… that the anisotropic growth of this and related highly polar acentric materials arises from intrinsic mechanistic causes …’ (Srinivasan & Sherwood, 2011 ▸).

Molecular dynamics simulations to investigate the melt (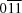) (fast-growing; hydroxyl groups) and melt (011) (slow-growing; H atoms) interfaces came to the conclusion that the slower growing face ‘… exhibits a weaker ability to direct and align the α-resorcinol molecules with the lattice …’ and ‘… the presence of rogue *C*_2_ conformers, which show some selectivity for incorporation into the emerging crystalline layer at this face …’ (Ectors *et al.*, 2015 ▸) may introduce further inhibition. In that sense a kind of ‘self-poisoning’ is responsible for retardation.

Although progress has been achieved for a representative crystal, we still do not have enough experimental and theoretical data for a general conclusion. Two further examples, *i.e.* of 2-cyclooctylamino-5-nitropyridine (COANP, *mm*2; not growing from the negative side, where nitro groups appear at the surface; undergoing 180° twinning on the negative side if supercooling occurs in the melt) and *meta*-nitroaniline (*m*-NA, *mm*2; growing in the gas phase at comparable speed along both directions of the axis 2), together with α-resorcinol, evidently set up a contradiction among these three materials concerning a preference for one type of charged face to grow or not to grow.

As mentioned above, polar crystals undergo charge compensation. Depending on the growth medium (vapor, solution, melt) the availability of free carriers is different. It could well be that the kinetics of processes on a surface can be influenced by the type, mobility and concentration of free charges.

### 1.2. Material types (ii) and (iii): biomimetic composite materials and biological tissues   

*In vitro* and *in vivo* composite materials made of a dipolar chiral biopolymer and a mineral can show polar alignment of the organic part, whereas the inorganic lattice does not contribute to the polarity. In metabolic systems, polar alignment on a large scale enables living creatures to process stimuli from the outside world (heat or pressure; Lang, 2000 ▸), whereas individual cells receive stimuli from their nearest environment (within a vicinity of about 100 µm). *In vitro* made materials may serve here to study model systems of reduced chemical and biological complexity (Kniep & Simon, 2007 ▸).

### 

This review is organized as follows. In Section 2 we will summarize the main principles of stochastic polarity formation elaborated for molecular crystals, and in Sections 3 and 4 we will address the findings for biomimetic composites and biological tissues, respectively. In Section 5 a comprehensive conclusion is given.

## 2. Effects of growth-induced stochastic polarity   

In the solid state, molecules can undergo a variety of structural disorder. Kitaigorodsky (1984 ▸) was one of the first to investigate 180° orientational disorder in molecular crystals. Recent work has applied a symmetry-adapted ensemble approach and force-field methods to study such defects in organic crystals (Habgood *et al.*, 2011 ▸).

In the years 1995–1998, 180° orientational disorder of dipolar entities was identified as a source of: (i) creating polar properties during growth upon a centric or acentric (but not polar) seed structure; or (ii) modifying the polar state of a crystal growing upon a polar seed structure (for references, see below). The basic principle behind (i) and (ii) is as simple as the following:

When a building block carrying an asymmetric charge distribution enters a site at a slow-growing face (for the theoretical description this means formally in the limit of no supercooling or supersaturation), there are crystal structures which allow for its incorporation by a 180° faulted orientation. Such a defect is associated with an endothermic change in the attachment energy Δ*E* = *E*_defect_ − *E*_normal_, but yields a positive Δ*S* for configurational entropy. This local two-state equilibrium (faulted *versus* normal) follows a Boltzmann distribution, a result obtained in analogy to the calculation of the concentration of Schottky defects (vacancy *versus* occupied) (Hulliger *et al.*, 2001 ▸). Assuming an equilibrium concentration of defects for the growth steps to follow (for layer-by-layer, edge or kink growth), this model can account for a progressive alignment of dipoles pointing in the same direction (Hulliger *et al.*, 2002 ▸; Wüst & Hulliger, 2007 ▸).

**Figure 2:**
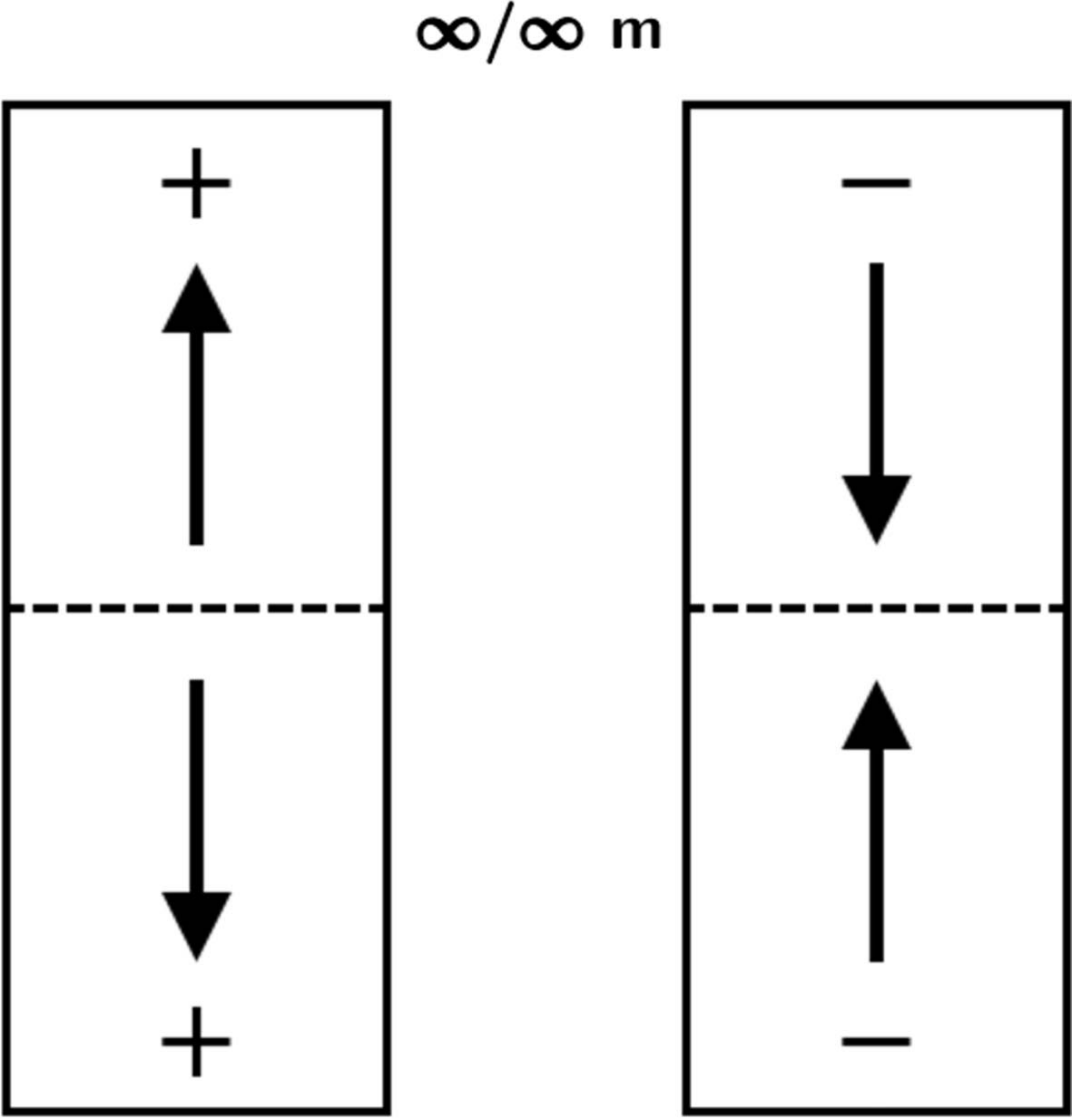
Bipolar macro states of solid matter. The object is built of two domains showing opposite polarities, yielding zero in total. The symbol of the continuous group for the entire object is ∞/∞*m* (equivalent symbol ∞/*mm* from *International Tables for Crystallography*; Authier, 2006 ▸).

Here, we encounter a breaking of symmetry at the crystal–nutrient interface, irrespective of the space group of the seed. In terms of the most frequent space group for molecular crystals, *P*2_1_/*c*, point group 2/*m*, the growing system loses, sector-wise, the mirror plane *m*. The symmetry 2/*m* is, however, preserved, but only at the level of the entire object. The net polar alignment of dipolar entities in either sector consequently shows an antiparallel relation. We call such a state ‘bipolar’ (see Fig. 2 ▸), following the notation of Shubnikov *et al.* (1955 ▸). Whenever possible, a thermally driven system will restore local symmetry breaking at a macroscopic level. In a more general context, this involves restoring of ‘ergodicity’ (Sethna, 2006 ▸).

The most instructive class of crystals for which growth-induced polarity formation has been experimentally demonstrated and theoretically explained by a Markov chain process (for mathematical details of Markov chains, see Zachmann, 1994 ▸) are channel-type inclusion compounds, which take up dipolar molecules in parallel channels (Hulliger *et al.*, 1995 ▸, 1997 ▸; König *et al.*, 1997 ▸; Harris & Jupp, 1997*a* ▸,*b* ▸). Over the years, analytical theory and Monte Carlo (MC) simulations have worked out a general framework for stochastic polarity formation, which is able to explain the observed effects for all three types of material (i)–(iii) we have mentioned above (Bebie *et al.*, 2002 ▸; Hulliger *et al.*, 2002 ▸).

To avoid possible misunderstanding, we should mention that the present theory does not provide mechanisms for the kinetics of growth in relation to polarity. Therefore, slow growth is assumed for comparison with the experimental data. We also have to emphasize that we assume kinetically stabilized faulted orientations when overgrown. Furthermore, we do not model the formation of a nucleus: the present description starts upon an already existing seed. However, we have investigated the states of seeds which can undergo 180° orientational disorder in the volume. These results make clear that, at the level of a nano-sized seed, a bipolar state may form as well (Hulliger *et al.*, 2013 ▸).

When searching for the origin of Δ*E*, we find that the dipole–dipole interaction between two molecules *i* and *j* (*i* = incoming and *j* = located within the surface) will yield Δ*E* = 0. The most relevant low-order pair of terms following from a multipole series describing the coulombic part is the dipole–quadrupole interaction *E_ij_* (Cannavacciuolo & Hulliger, 2016 ▸). Additionally, a Lennard–Jones-type potential ensures contributions to non-zero Δ*E* values.

When calculating Δ*E* by force-field methods, it was seen that summation over the next-nearest neighbors *j* was already sufficient (Gervais *et al.*, 2005 ▸) to obtain a nearly converged value for Δ*E_ij_* (*j* = 1, …, *n*). Depending on the crystal structure, the number of energetically different surface sites and the number of neighbors *n*, the analysis will have to take into account Δ*E_ij_* values for all corresponding sites at each of the (*hkl*), (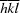) surfaces.

Summarizing, we can say that 180° orientational faults occurring along the growth of a centric or acentric (but not polar) seed can produce corresponding sectors where physical methods (SPEM, PFM, PS-SHM) allow us to visualize a spatially inhomogeneous distribution of the bulk polarity. Here, the combination of SPEM and PS-SHM with Bijvoet experiments (anomalous X-ray scattering, sector-wise Flack parameter analysis) has worked out the details of the real polar structure of molecular crystals (Burgener *et al.*, 2013 ▸).

In view of this theoretical and experimental work, we can conclude that, in principle, all as-grown molecular crystals made of dipolar building blocks may show polar effects in particular sectors [for an analysis of (*hkl*) faces undergoing polarity formation, see Gervais & Hulliger, 2007 ▸]. The strength of the normally weak grown-in polarity, however, depends on Δ*E* and the corresponding probabilities *P*_defect_. Clearly, not every crystal packing can easily accommodate 180° inverted building blocks at surface sites or in the bulk.

**Figure 3:**
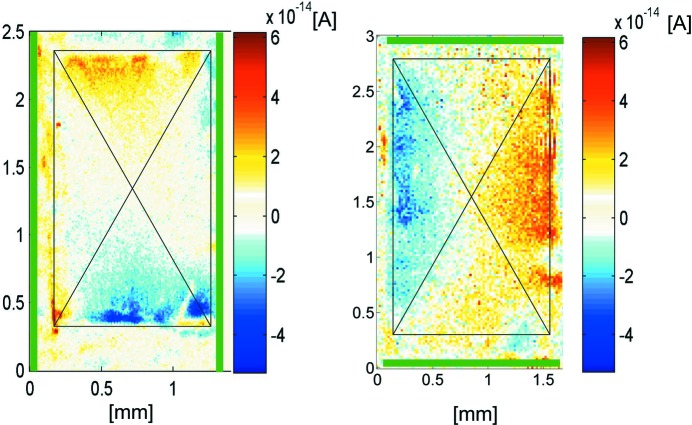
SPEM data (Burgener, 2014 ▸) for an NaClO_3_ crystal. Electrodes (green) measure the pyroelectric current in the [001] and [010] directions. The crystal was obtained by isothermal evaporation (25°C) of water. Alternating polarity appears in opposite sectors. Due to structural features, here the polarity is detected at 90° to the electrodes (normally at 0°).

An example from the class of ionic crystals containing dipolar molecular units (sodium chlorate, point group 23) illustrates our conclusion: SPEM measured for solution-grown NaClO_3_ reveals weak polarity in sectors for all cubic directions {100} (Fig. 3 ▸). We stated above that orientational faults are assumed to be kinetically stabilized at a temperature far below melting. The example of NaClO_3_ allows us to investigate the thermal behavior: polarity grown-in at room temperature disappears when the crystals are heated, *e.g.* up to 235°C for 7 d (Burgener, 2014 ▸) (melting point 255°C).

We now proceed to a discussion of the growth behavior of polar seeds.

**Figure 4:**
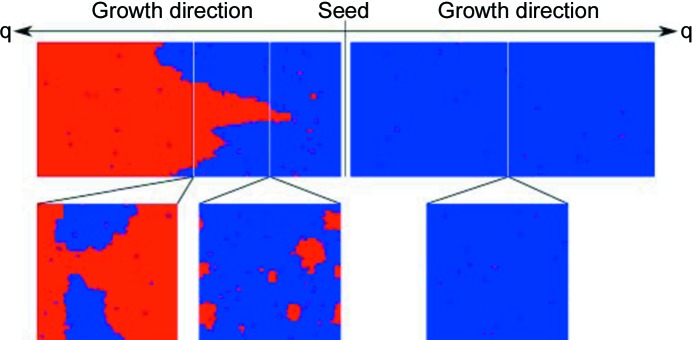
Snap shots of Monte Carlo simulations for a layer-by-layer growth model. Along one side of the polar axis, complete reversal takes place after some advancement (Hulliger *et al.*, 2013 ▸). For more details, see text.

MC simulations for an anisotropic two-dimensional Ising-type model including nearest-neighbor interactions within a square lattice (Bebie *et al.*, 2002 ▸; Hulliger *et al.*, 2002 ▸) have led to the discovery of the so called ‘reversal transition’. In Fig. 4 ▸ we show the MC advancement of a layer-by-layer growth mode, starting from a mono-domain polar seed (middle). Layers are attached to both sides of the polar axis. Towards the right-hand side some single defects or small clusters (in red) may appear and disappear within a mostly homogeneous sector (in blue). Towards the left-hand side some advancement (blue) occurs as well, but there are more and larger clusters in red. Suddenly, such a cluster starts to expand into a cone, ultimately producing a nearly homogeneous but polarity-inverted red sector. This kind of growth-induced transition is seen in MC simulations for (i) layer-by-layer, (ii) edge and (iii) kink growth modes (Wüst & Hulliger, 2007 ▸). In the case of the kink (iii), the number of growth steps is lowest to start the transition and to complete the reversal. Simulations also show that the transition can start from a single defect coming up at a density of as low as a few percent (Hulliger *et al.*, 2001 ▸). On which side the reversal preferentially occurs depends on the Δ*E* and *P*_defect_ values calculated for the corresponding (*hkl*) and (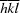) faces.

A probabilistic model for the formation of clusters showing an inverted polarization supports a ‘critical size’ effect. In the case of the layer-by-layer growth mode, clusters of a rather small size (*n* × *n* entities) of 4 × 4 (7 × 7 is the maximum of the size distribution) can grow further by a probability of nearly 1. This applies to the direction for which MC simulations (Fig. 4 ▸) find reversal. In the opposite direction, larger clusters are required (smallest 7 × 7, maximum 9 × 9, the distribution being very broad towards large clusters), which renders reversal less probable. These estimations, however, depend on the parameter space of the possible intermolecular energies *E_ij_* that we have randomly explored.

In this context we can demonstrate that the reversal transition cannot be properly described by a Markov chain process. It is best represented by a two-dimensional nucleation phenomenon.

**Figure 5:**
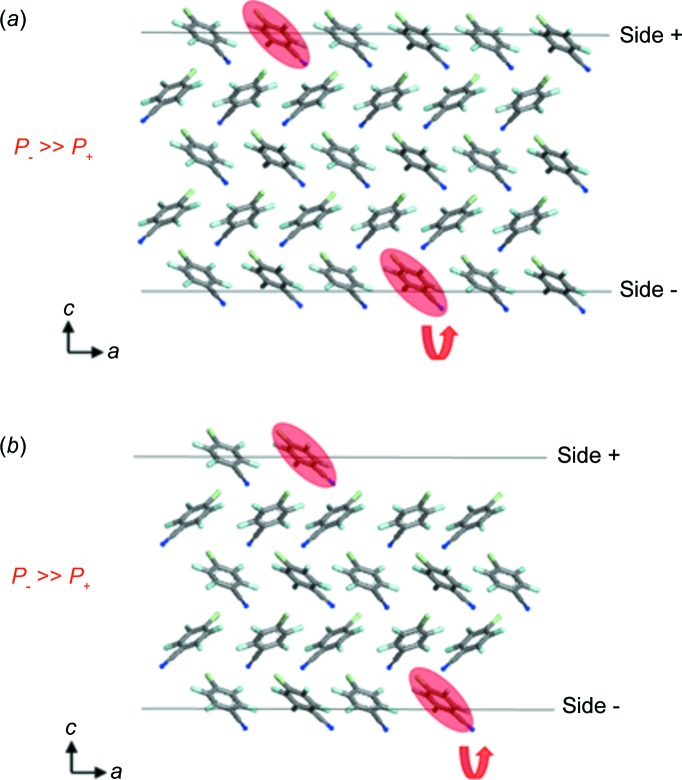
Force-field based prediction for starting reversal on (*a*) (001), (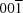) flat faces and (*b*) kink sites. 1-Chloro-4-cyano-tetrafluorobenzene molecules which may undergo 180° faults are marked. The curved arrow indicates their reversal. *P*_−_ > *P*_+_ means that reversal should start at the cyano face (Brahimi & Hulliger, 2016 ▸).

A rather simple case will serve here as a numerical example of 180° defect formation (Brahimi & Hulliger, 2016 ▸). The structure of 1-chloro-4-cyano-tetrafluorobenzene (*Pca*2_1_, *mm*2; Bond *et al.*, 2001 ▸) provides only one site per alternating surface layer for (001), (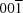) faces. Using the universal force field and Gasteiger charges, we have calculated the probabilities (*P*_defect_, 300 K) *P*_+_ and *P*_−_ (+ denotes the positive *c* direction of axis 2 with the surface covered in chloro groups, and correspondingly − denotes the negative direction with the surface covered in cyano groups) to form a 180° inverted attachment at (i) flat (001), (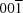) faces and (ii) kink sites. Schematic views of the structure are shown in Fig. 5 ▸. Here, *P*_−_ = 0.4 is clearly larger than *P*_+_ = 0.09. This means that the initial process for reversal (primary defect formation; Hulliger *et al.*, 2001 ▸) should take place from the cyano side. Following our previous analyses (Hulliger *et al.*, 2002 ▸) based on decomposition of the interactions within a lattice into longitudinal [functional-group interactions such as donor–acceptor (*D*⋯*A*), donor–donor (*D*⋯*D*) and acceptor–acceptor (*A*⋯*A*); Desiraju, 1995 ▸] and transverse (generally larger cross section; Gervais *et al.*, 2005 ▸) contributions, we notice that in this structure the molecules do not build up chains but make CN⋯π contacts. Therefore, it is not surprising that reversal could start on the cyano side, whereas for typical chain-forming structures (due to *A*⋯*D* synthons; Desiraju, 1995 ▸) reversal is predicted for the donor side. For this crystal, there is as yet no morphological (grown from the gas phase), Bijvoet and SPEM analysis to provide a comparison with the theoretical result.

MC simulations and force-field calculations let us conclude that polar seeds can show a certain probability of developing into a macroscopic two-domain state, previously introduced as bipolar (Hulliger *et al.*, 2012 ▸). Ideally, the crystal will show no macroscopic dipole moment in its final growth state (Fig. 2 ▸).

At this stage we recognize the demand of a quantum statistical statement: in the stationary state a system does not show an electric dipole moment (Anderson, 1972 ▸). By ‘stationary’ we understand here that the system has exceeded thermalization with respect to one degree of freedom that, for our discussion, is 180° orientational disorder.

The far-reaching consequence of this is that a macroscopic mono-domain state of polar molecular crystal structures may not represent the most likely state. A stationary state can be bipolar. This is clearly represented by nanometre-sized seeds undergoing 180° orientational disorder in their bulk (Hulliger *et al.*, 2013 ▸). A similar behavior is well known for ferroelectric crystals: they split into a domain state (Blinc, 2011 ▸), albeit by a different mechanism.

At this point we should add a comment on polar crystal structures found for centric molecules. These cases express geometric polarity, although the crystal field will lower the symmetry of the molecules, thus producing weak effects of lattice polarity. However, during growth there is no accessible degree of freedom for these systems which could lead to a bipolar growth state. It would be of great interest to investigate the growth behavior of such crystals along their polar axis. Here, the effect of electrostatic interactions is much smaller than the influence dipolar building blocks can have.

Evidently, there seems to be a contradiction to data reported in the CSD and to literature on morphological and physical descriptions of molecular crystals. Well characterized mono-domain examples provide, however, no basic argument against a non-zero probability of forming a bipolar state. The existence of mono-domain crystals brings us back to the phenomenon of anisotropic growth: a close-to-zero growth speed along one direction of the polar axis may be related to 180° defect formation, leading to kinetic hindrance for these faces (self-poisoning effect, see Section 1). Because of a certain, though small, probability of reversal we can find here a common base for polar molecular crystals to undergo a particular kind of self-poisoning. In that sense, *P*_defect_(*hkl*), *P*_defect_(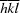) values could open up a perspective to predict which side may show slow or even no growth.

**Figure 6:**
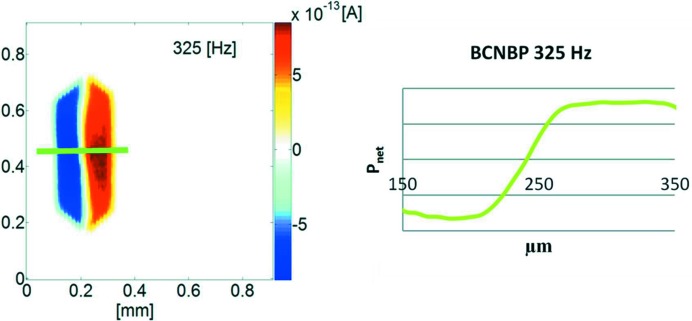
SPEM data for a polished *bc* plane of a 4-bromo-4′-cyano-biphenyl (BCNBP) crystal. (Left) A two-dimensional scan over the plane, showing two domains of opposite polarity separated by a transition zone. (Right) A pyroelectric scan to investigate the transition zone extending over a width of about 100 µm, where the polarization reaches zero to become inverted (Burgener *et al.*, 2016 ▸).

Recently, we presented an initial experimental demonstration of a reversal transition (Burgener *et al.*, 2013 ▸). The low-temperature polymorph of 4-iodo-4′-nitro-biphenyl (INBP) (crystallizing in *Fdd*2, *mm*2) expresses a nearly octahedral (*i.e.* symmetric) morphology (solution growth in 2-butanone). The observed habitus is in clear contradiction of a single domain state in *mm*2. Here, SPEM and the measurement of the Flack parameter in each sector have clearly worked out a bipolar state. The transition zone where the local polarization changes its sign spans a distance of about 150 µm, an indication that classical twinning can be excluded. Meanwhile, other examples (Fig. 6 ▸) have demonstrated such kinds of behavior (Burgener *et al.*, 2016 ▸).

**Figure 7:**
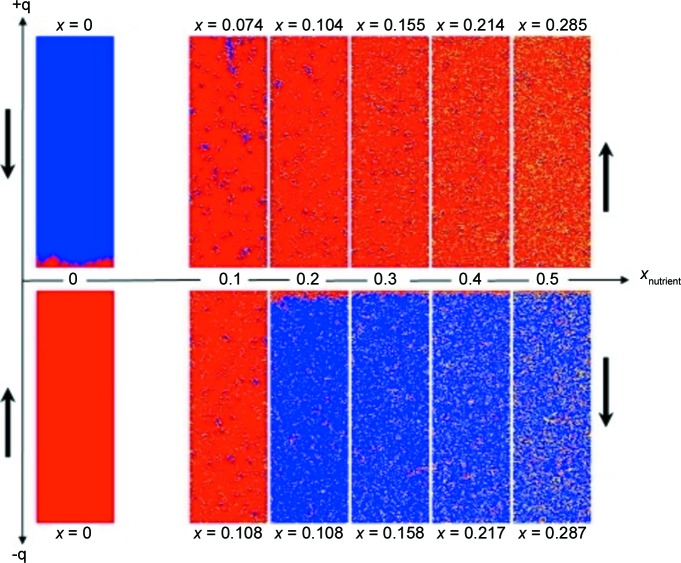
Monte Carlo simulations demonstrating the effect of symmetric biphenyls (*e.g.* 4,4′-diiodo-biphenyl, DIBP) on polarity formation of an asymmetric analogue, *e.g.* 4-iodo-4′nitro-biphenyl (INBP). At zero content (*x*_nutrient_) of the symmetric component, reversal occurs in the upper part (see left-hand side). Addition of the symmetric component to the nutrient kept a low concentration favors at first a mono-polar state which for higher *x*_nutrient_ changes into an inverted state compared with the initial reversal (Hulliger *et al.*, 2014 ▸).

Essential support for a stochastic reversal-type mechanism was obtained by growth experiments where symmetric 4,4′-disubstituted donor-type biphenyls were added to the nutrient. For such a system, MC simulations predict an inverted bipolar state due to their presence in the nutrient, along with a small uptake of symmetric components (solid solution, see Fig. 7 ▸). This means that the two-component system produces corresponding domains where the polarization is inverted compared with the one-component case (Hulliger *et al.*, 2014 ▸). Currently, this effect is experimentally proven for three types of real *A*–π–*D* biphenyls (Burgener *et al.*, 2013 ▸, 2016 ▸), but it was first observed for channel-inclusion compounds (Roth *et al.*, 1998 ▸; König *et al.*, 1997 ▸). Furthermore, MC simulations demonstrate the promotion of primary reversal due to the addition of acceptor-type symmetric components.

Summarizing, we can say that theoretical predictions of stochastic polarity formation are experimentally proven by an increasing number of real as-grown molecular crystals.

## 3. Bipolar state of inorganic macromolecular composite materials   

Composite materials formed in gels made of a biogenic mineral and macromolecules are of interest for the study of *in vitro* processes which may serve as a model to understand the formation of *in vivo* hard tissues.

Long-term research by the group of Kniep & Simon (2007 ▸) on the formation of fluoro­apatite (FAP) in gelatin has elaborated a detailed view of the processes leading to a composite solid (mineral and about 2 wt% organic material) expressing a prismatic seed at the early stage of growth and developing further into a dumb-bell shaped or quasi-spherical final object.

An SPEM analysis has recently demonstrated (i) a mono-domain polar seed state and (ii) a bipolar dumb-bell growth form. The analysis allowed us to conclude that, in the second growth phase, the N-termini of the collagen helices are mostly aligned in the direction of growth. Because a final FAP object is bipolar and the seed represents a mono-domain state, we find here also a growth-related reversal of polarity (Burgener *et al.*, 2015 ▸).

**Figure 8:**
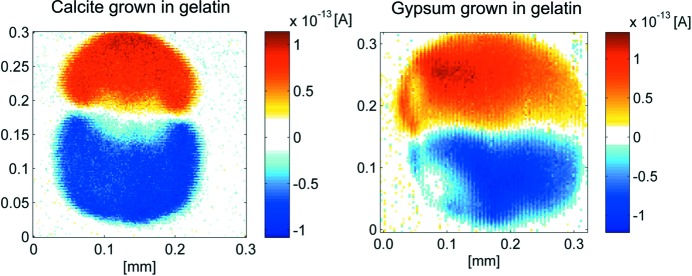
Polarity distribution for polished (down to the middle of the quasi-spherical sample) dumb-bell objects, grown in a gel made of gelatin (10 wt%) and water. Both objects are bipolar and show the same pyroelectric current flow direction as fluoro­apatite (FAP; Burgener *et al.*, 2015 ▸). The result also shows that, for these cases, the N-termini point towards the growing interface (Sommer *et al.*, 2016 ▸; extended paper to be published).

Initially, dumb-bell type growth and bipolarity were only observed for the FAP system. Recently, we have extended the analysis (Sommer *et al.*, 2016 ▸) to other minerals [CaSO_4_ (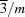), CaCO_3_ (2/*m*) and CaC_2_O_4_ (2/*m*), as well as further examples] and other macromolecules (*Agar agar*, carrageenan). To our great surprise, all these systems show (i) a dumb-bell type growth form and (ii) a bipolar state (Fig. 8 ▸). These data let us conclude that, for axial point groups of minerals, cations such as Ca^2+^ serve to align and bundle the helices to promote the growth of a composite object also featuring axial symmetry.

## 4. Polarity formation in natural tissues and by cells   

Natural tissues are organized in a hierarchical manner (for an introduction, see Bilezikian *et al.*, 2008 ▸). Along their growth, tropocollagen monomers aggregate into fibers (Kadler *et al.*, 1996 ▸). The final fiber pattern is of fundamental importance for the occurence of mechanical and polar properties.

The discovery of piezoelectric and pyroelectric properties in natural tissues (bones, tendons, nerves *etc.*) dates back to the years 1966–1967. At that time it was anticipated that these electric signals induced by external stimuli (pressure, heat) might contribute to the basic functions of living creatures (Lang, 2000 ▸).

Because of the homochiral property of mainly type II collagen, a side-to-side alignment of helices, as found in particular parts of tissues, produces a piezoelectric material showing only shear tensor elements (*d_ijk_*) being non-zero. The continuous average point group is ∞2. To obtain a pyroelectric response, nature must involve an orientational preference for collagen building blocks elongating the fibers. In 2003 we presented the first theoretical model which could explain bio-grown polarity (∞ group) by a Markov chain mechanism, making use of biochemical knowledge of the functional-group interactions of the N- and C-termini of the helices (Hulliger, 2003 ▸). The Markov model, combined with biological information (Kadler *et al.*, 1996 ▸) on fiber elongation by fibroblasts, allowed us to conclude that the C-termini are oriented in the direction of biological growth, called distal. The main thrust of our experimental analysis was then to elaborate the local orientation of polarity in tissues and compare the data with theoretical predictions. SPEM data for cortical bone (mouse) and a negative pyroelectric coefficient [calculated by a molecular dynamics simulation for a model collagen helix (Ravi *et al.*, 2012 ▸)] support C-termini aligned in the direction of biological growth for that type of bone (Burgener *et al.*, 2015 ▸). As elongated thigh bone (femur) grows from a central part in two directions, separate domains of opposite polarization build up, *i.e.* a bipolar state is observed.

**Figure 9:**
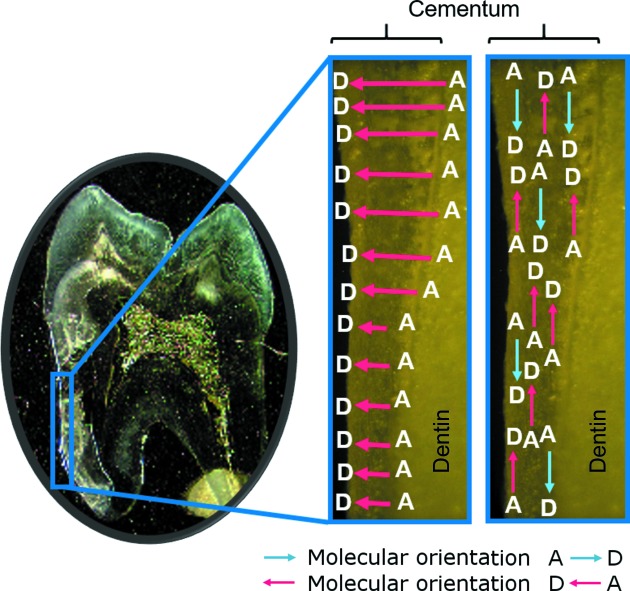
Polarity distribution in a human tooth (for more details, see Aboulfadl & Hulliger, 2015 ▸), measured by absolute phase-sensitive second-harmonic microscopy (PS-SHM) using a nonlinear optical reference crystal. (Left) An area near the surface showing mono-polar alignment of collagen fibres in cementum. Here, donor (D) groups are preferentially oriented towards the surface. (Right) An area of more distributed or bipolar alignments in cementum.

Further work in this context has applied PS-SHM (absolute polarity determination by use of a polar reference crystal; Aboulfadl *et al.*, 2013 ▸) to provide a two-dimensional map of the absolute polarity distribution in *e.g.* cementum of human teeth (Aboulfadl & Hulliger, 2015 ▸). Here, a mono-domain state was found for acellular extrinsic cementum. In contrast, in the circumferential direction two corresponding domains were observed featuring an opposite sign of polarity, indicative of a bipolar microscopic state of intrinsic cellular cementum. From the absolute phase experiment we can conclude that the orientation of radial collagen fibers is organized to show N-termini preferentially at the surface (Fig. 9 ▸). Here, biological investigations will be needed to show from which side the fibers are elongated, in order to compare the PS-SHM result with a Markov model.

The growth of tropocollagen monomers into fibers and their general alignment within tissues are the result of complex processes regulated by cells. One can distinguish areas of large- and small-scale mono-polar and bipolar alignments. In particular, domain formation on the micrometre scale is poorly understood. Here, the application of SPEM, PFM and the absolute PS-SHM technique open up new perspectives for investigating tissue formation and diseases related to a mis­alignment of collagen fibers.

Up to this point we have discussed the effects of growth-induced polarity along a series of systems showing an increasing level of complexity. To end this section, we would like to comment on the polar geometric organization of cells. Because of polar cellular components, geometric cell polarity also expresses electric polarity.

Cells in organs from yeast to humans contrive to arrange and maintain an asymmetric spatial distribution of functional components, called cell polarity. During the establishment and maintenance of cell polarity, polarity complexes, *i.e.* proteins, interact with each other. Polarity proteins are key regulators for microtubules (showing about 95% polar alignment of building blocks) and the dynamics of the Golgi apparatus. The polar structure of cell components allows for signaling cascades and the directed transport of species through membranes and along microtubules. All these processes need a high degree of regulation. Dysregulation of cell polarity can cause developmental disorder and may promote cancer (for a review, see Muthuswamy & Xue, 2012 ▸).

## 5. Summary and final conclusions   

In view of the many aspects discussed above, we shall conclude with a principle unifying the current knowledge of polarity formation for material systems (i)–(iii).

**(i) Crystals.** At the lowest level of the hierarchy, polarity finds its origin by joining atoms of different electronegativities. By a series of chemical or biochemical reactions, larger building blocks emerge. At this stage, kinetic control may yield centric/acentric or polar seed structures of molecular crystals. From there, growth can transform them into a bipolar state featuring typically weak polar effects, or undergo either anisotropic growth by self-poisoning or a reversal transition to yield strong polar effects in both of these cases.

**(ii) Composite materials.** Here, cations aligning helices are at the basis of an organization which may lead to polar order. During growth, polarity formation in each hemisphere of a dumb-bell is driven by functional-group interactions, also promoting a reversal in cases where the seed is mono-polar. A bipolar final growth state is typically observed.

**(iii) Tissues.** At the level of biological growth, elongation of aligned fibers produces a net polar order, driven by the effects of the recognition of N- and C-termini.

To find C-termini preferentially in the direction of bio­logical growth of bones and N-termini at the interface of a composite is, in both cases, in agreement with the Markov chain prediction using knowledge of the interaction of termini in helices. The difference in the observed orientation is due to the mechanism of elongation: *in vivo* fibers can be assembled from the back (intracellular), whereas *in vitro* alignment occurs from the front.

For systems (i) to (iii) we recognize a unifying principle: the formation of polarity requires a non-zero difference in the probabilities for 180° inverted surface states of building blocks, and uni-directional growth. For systems (i)–(iii), a Markov-type process in the first instance produces polar domains (large or small).

The bipolar state may just be a consequence of growth along opposite directions (for crystals, composites and tissues). However, for a mono-polar origin of growth, crystals, composites and even tissues may show a reversal transition because of an extension in both directions of the polar axis. The bipolar state also applies to seeds which can undergo 180° disorder. The mechanism of the reversal transition is based on a critical size for the initial clusters formed through fluctuations.

Finally, addressing cell polarity we ask whether the principles we have discussed here may find application even at the level of cells. In view of the generality of Markov chains, we might think that recognition processes between species in cells could provide a base for conditional probabilities driving their geometric polar organization.
